# Genome-Wide Transcriptional Analysis Reveals Alternative Splicing Event Profiles in Hepatocellular Carcinoma and Their Prognostic Significance

**DOI:** 10.3389/fgene.2020.00879

**Published:** 2020-08-11

**Authors:** Yongfu Xiong, Gang Yang, Kang Wang, Muhammad Riaz, Jian Xu, Zhenbing Lv, He Zhou, Qiang Li, Weinan Li, Ji Sun, Tang Tao, Jingdong Li

**Affiliations:** ^1^Department of Hepatobiliary Surgery, Affiliated Hospital of North Sichuan Medical College, Nanchong, China; ^2^North Sichuan Medical College, Institute of Hepato-Biliary-Pancreatic-Intestinal Disease, Nanchong, China; ^3^Department of Breast Surgery, The First Affiliated Hospital of Chongqing Medical University, Chongqing, China; ^4^Department of Gastrointestinal Surgery, Nanchong Central Hospital, Nanchong, China; ^5^Department of Gastrointestinal Surgery, Affiliated Hospital of North Sichuan Medical College, Nanchong, China

**Keywords:** hepatocellular carcinoma, alternative splicing, genome-wide, RNA-seq, prognosis

## Abstract

Accumulating evidence indicates an unexpected role of aberrant splicing in hepatocellular carcinoma (HCC) that has been seriously neglected in previous studies. There is a need for a detailed analysis of alternative splicing (AS) and its underlying biological and clinical relevance in HCC. In this study, clinical information and corresponding RNA sequencing data of HCC patients were obtained from The Cancer Genome Atlas. Percent spliced in (PSI) values and transcriptional splicing patterns of genes were determined from the original RNA sequencing data using SpliceSeq. Then, based on the PSI values of AS events in different patients, a series of bioinformatics methods was used to identify differentially expressed AS events (DEAS), determine potential regulatory relationships, and investigate the correlation between DEAS and the patients’ clinicopathological features. Finally, 25,934 AS events originating from 8,795 genes were screened with high reliability; 263 of these AS events were identified as DEAS. The parent genes of these DEAS formed an intricate network with roles in the regulation of cancer-related pathway and liver metabolism. In HCC, 36 splicing factors were involved in the dysregulation of part DEAS, 100 DEAS events were correlated with overall survival, and 71 DEAS events were correlated with disease-free survival. Stratifying HCC patients according to DEAS resulted in four clusters with different survival patterns. Significant variations in AS occurred during HCC initiation and maintenance; these are likely to be vital both for biological processes and in prognosis. The HCC-related AS events identified here and the splicing networks constructed will be valuable in deciphering the underlying role of AS in HCC.

## Introduction

Despite great advances in recent decades in screening, diagnosis, and curative surgery, hepatocellular carcinoma (HCC) remains the second leading cause of cancer-related mortality worldwide (Grandhi et al., 2016; Siegel et al., 2018). Epidemiological evidence has confirmed that the long-term outcomes of patients with HCC have not improved significantly with the rapid development of surgical techniques (Madduru et al., 2019). More importantly, because of the limitations of systematic status, tumor position, and the need to preserve liver function, more than 70% of patients are not eligible for surgical treatment. Even after curative resection, prognosis remains unsatisfactory because of a high incidence of postoperative recurrence (Colecchia et al., 2014). The initiation and maintenance of HCC is a complex and regulated process involving the accumulation of numerous genetic changes over decades (Niu et al., 2016; Erkekoglu et al., 2017). These sequential alterations not only endow normal liver cells with neoplastic ability, enabling uncontrolled growth, but also provide potential therapeutic targets and biomarkers. Thus, further understanding of the initiation and maintenance of HCC at the molecular level is crucial to prolonging survival and making individual treatment decisions.

The explosive development of high-throughput technology has provided powerful tools for the molecular study of cancer (Schmidt and Hildebrandt, 2017). RNA sequencing (RNA-Seq) and microarrays, the most representative methods of this technology, are mature enough for use in commercial applications (Mantione et al., 2014; Zhang et al., 2015). During the past decades, the genome-wide transcriptional analysis of gene expression has become critically important to gain better insight into the biological processes of HCC and other types of cancer (Jin et al., 2015; Xiong et al., 2017). In addition to the aberrant expression of transcripts, studies have focused on different molecular levels (multi-level omics), including copy number variation, epigenetic modifications, nucleotide polymorphisms, and DNA methylation, especially in HCC (Lee et al., 2016; Lin et al., 2017). Evidence obtained from these studies clearly demonstrates that HCC is a disease caused by cumulative aberrations at different levels of molecular regulation; thus, only a high-throughput multi-omics analysis can decipher the complex biology of HCC. Many previous studies, despite promising results, focused only on the aberrant regulation of expression and its biological effects. However, structural transcript variation in HCC, which is heavily shaped by alternative splicing (AS), has until recently been less well studied.

According to the manual genome annotation project (Harrow et al., 2012; Pruitt et al., 2014), there are only about 20,000 protein-coding genes. This number is obviously inconsistent with the overall cellular complexity, which includes at least 82,141 distinct protein-coding sequences (Harrow et al., 2012). This discrepancy between the numbers of transcripts and protein-coding genes in human cells indicates the existence of an additional mechanism, between the transcriptional and the post-translational levels, that increases the coding capacity of the genome. Through the AS process, a single RNA precursor can be spliced *via* distinct arrangements to generate RNAs with different structures and functions (Biamonti et al., 2014; Song et al., 2018). This may be one of the main causes of cellular complexity and proteome diversity. Experimental studies on the effects of individual AS events suggest that AS may change the biological function of a protein by regulating its stability, controlling its location, modifying the mutual interactions of proteins, and even adding or deleting active domains (Brett et al., 2002; Yang et al., 2016). These findings suggest that, as well as expression abundance, the balance of different AS events that result from the same RNA precursor must be considered. However, the latter consideration has often been neglected in previous studies. In fact, emerging data from genome-wide analyses (Feng et al., 2013; Kahles et al., 2018) indicate that AS occurs in more than 95% of multi-exon genes, suggesting that the widespread existence of AS is the product of physiological processes rather than transcription errors.

In recent years, the diagnostic and the therapeutic role of AS in many human diseases has attracted increasing attention. Large-scale screening of AS events has been performed using expressed sequence tag libraries, although this approach is prone to a high rate of false positives (Venables, 2004). Exon junction probes provide a higher experimental validation rate (Lapuk et al., 2010). However, this method has the disadvantage of being limited to known splice junctions. Owing to the limited available techniques, complicated mechanisms, and huge numbers involved, transcriptome-wide AS dysregulation and its potential associations with biological behavior in HCC have remained uncharacterized.

RNA-Seq not only supports the quantitative measurement of novel AS events but also provides deeper coverage, higher accuracy, and better resolution (Li Y. et al., 2017); thus, it may be the most suitable of the currently available approaches for AS study. In recent years, The Cancer Genome Atlas (TCGA) (Tomczak et al., 2015; Wang et al., 2016) has accumulated a rich and publicly available source of RNA-Seq data and corresponding clinical information. This enables the analysis of AS dysregulation in HCC at a genome-wide level. TCGA includes 415 RNA-Seq data samples obtained from 365 HCC patients, together with their corresponding clinical information, thereby facilitating the clinical analysis of HCC-related AS events in a large cohort. However, without reliable and efficient bioinformatical methods, the advantages of RNA-Seq in AS analysis cannot be fully exploited. SpliceSeq, a recently developed bioinformatics tool, can exactly match RNA reads with gene splice graphs and is helpful for accurately calculating complex or low-frequency AS events (Ryan et al., 2012).

There has been a lack of studies combining large-scale RNA-Seq data with the corresponding clinical information to comprehensively analyze AS at single-exon resolution; however, this is very necessary, especially in HCC. Therefore, in the current study, we comprehensively analyzed whole-genome AS in the TCGA HCC cohort to screen out HCC-related AS events and further studied the relationships of these events with clinical outcomes. Our findings suggest that certain HCC-related AS events, including NEK2-AT and TROPT-AT, have critical roles in the progression and maintenance of HCC. More importantly, these HCC-related AS events represent potential new therapeutic targets.

## Materials and Methods

### Data Curation

Clinicopathological information of the HCC cohort and corresponding RNA-Seq data were retrieved and downloaded from TCGA^1^. To ensure appropriate protection of patient privacy, the TCGA data were stratified according to data type and level, conforming to the publishing guidelines formulated by TCGA (Wang et al., 2016). Then, the RNA-Seq data and corresponding clinicopathological information were mutually paired using the unique TCGA barcodes. Only patients who met the criteria listed below were included: (Grandhi et al., 2016) patients with corresponding RNA-Seq data, (Siegel et al., 2018) patients with complete clinicopathological information, including local invasion, tumor location, sex, age, distal metastasis, pathological stage, differentiation grade, lymph node metastasis, and survival information, (Madduru et al., 2019) histological diagnosis of HCC, and (Colecchia et al., 2014) survival for at least 1 month after the primary pathological diagnosis. SpliceSeq was used to determine RNA splicing patterns and produce AS profiles for each HCC patient as previously described (Li Y. et al., 2017; Zhu et al., 2018). Each AS event was quantified using the percent spliced in (PSI) value (ranging from 0 to 1), a commonly used method to reflect the abundance of AS events. In order to remove the effects of splicing noise and generate as reliable a set of AS events as possible, a series of strict filters (average PSI ≥ 0.05, percentage of samples with PSI ≥ 75) was applied to the detected AS events. The interactive sets between the seven types of AS were quantitatively analyzed, and the results were visualized in UpSet plots using UpSetR (version 1.4.1) (Conway et al., 2017). Circlize (version 0.4.1) was used to generate circos plots to depict the parent genes and their AS events in chromosomes (Gu et al., 2014). The details of the design of the present study are illustrated in Supplementary Figure S1. All the methods used in this study were in line with the relevant guidelines and regulations.

### Identification of DEAS and Enrichment Analysis

To screen the differentially expressed alternative splicing (DEAS) events between HCC and corresponding normal tissues, the PSI value of each AS event was determined in the TCGA HCC cohort (371 HCC tissue samples and 50 paired adjacent normal tissues). A generalized linear model was applied to remove the batch effects. The DEAS were determined based on adjusted *P* (adj *P*) and associated log_2_ fold change (FC) values, with adj *P* ≤ 0.05 and |log_2_(FC)| ≥ 1 representing AS events that were downregulated and upregulated, respectively. Biological function enrichment analysis was performed based on the DEAS parent genes. Gene ontology (GO) and Kyoto Encyclopedia of Genes and Genomes (KEGG) terms with false discovery rate less than 0.05 were considered to be significantly enriched and were selected for further analysis. Enrichment analysis was performed using the ClusterProfiler package (version 3.10) (Yu et al., 2012). The parent genes of DEAS events were imported into the STRING 9.1 database and used to determine protein–protein interactions (PPIs). A relationship network was then generated using Cytoscape (version 3.7.2) (Su et al., 2014). Cluster analysis was conducted using the average linkage agglomeration algorithm and correlation distance metrics.

### Establishment of HCC-Related Splicing Correlation Network

A total of 71 splicing factors (SFs) (Supplementary Table S1) were identified by comprehensive and hand-curated screening of the literature. All the SFs included in the current study had been experimentally validated in previous research (Giulietti et al., 2013) and included 27 heterogeneous nuclear ribonucleoproteins proteins, 13 serine/arginine-rich proteins, and 31 other proteins belonging to the CELF, Fox, KHDRBS, Nova, and ELAV families. The expression of each SF was obtained from the BROAD institute^2^. Correlations between the PSI values of DEAS and the expression of SFs were analyzed by weighted gene co-expression network analysis (version 1.68) (Langfelder and Horvath, 2008). Benjamini and Hochberg correlation was used to adjust the *p*-values; the adjusted *p*-values less than 0.05 were considered to indicate statistically significant differences. Cytoscape (version 3.4.0) was used to generate the correlation plots.

### Survival Analysis

All the included HCC patients were divided into two groups based on the PSI value of each DEAS (median cut), and the two artificial categories were modeled as continuous variables to derive more easily interpretable hazard ratios. Based on overall survival (OS) and disease-free survival (DFS), Cox regression was performed to evaluate the prognostic value of each DEAS event. Log-rank test and Kaplan–Meier analysis were used to compare patient survival between subgroups; *p* < 0.05 was considered as statistically significant. The overall survival-related DEAS were further analyzed in LASSO regression to identity the most powerful prognostic markers. Finally, a prognostic model was constructed for predicting the OS. In order to quantify the risk of OS, a standard form of risk score (RS) for each colorectal cancer (CRC) patient was calculated. Combine the levels of the PSI (PSI*_*i*_*) and LASSO coefficients (*L*_*i*_), risk score = $\sum_{\iota =1}^{n}P\,S\,I\,i\times L\,i$, to divide the patients into the high- or low-risk group. Kaplan–Meier curves were used to estimate the survival for patients in the training, the testing, and the validation sets between the high-risk and the low-risk groups.

### Functional Experiment of CXCL12 Splicing Variants in HCC

The human HCC cell line HepG2 was obtained from the Chinese Academy of Sciences Committee on Type Culture Collection Cell Bank (Shanghai, China). The cell line was cultured in 1640 (Gibco, Carlsbad, CA, United States), supplemented with 10% fetal bovine serum (FBS; BI, Beit Haemek, Israel) at 37°C with 5% CO_2_. Total cDNA from tissues was obtained as described above. The PCR reaction was carried out using the forward primer 5′-tgcccttcagattgttgcac-3′, common for all isoforms, and the isoform-specific reverse primers 5′-gctaactggttagggtaatac-3′ and 5′-gctagcttacaaagcgccagagcagagcgcactgcg-3′ for NP_954637.1 and NP_001029058.1, respectively. b-Actin, amplified using the forward 5′-acactgtgcccatctagcagggg-3′ and reverse 5′-atgatggagttgaaggtagtttcgtggat-3′ primers, was used as a loading control. Quantitative real-time PCR was performed in Mx3005Tm QPCR System with a MxPro QPCR Software 3.00 (Stratagene, La Jolla, CA, United States) and SYBR Green detection system. The adherent HepG2 cells were transfected with the corresponding His-CXCL12 construct by the calcium phosphate method and cultured for 48 h. At 4 h before collecting them, the cell supernatants were removed and, when indicated, Brefeldin A was added to the fresh medium. The collected cells were left untreated or permeabilized with saponin and immunolabeled with the His mAb and a PE-goat anti-Ig secondary antibody and analyzed by flow cytometry. The cell invasion assay was conducted using Matrigel-coated chambers (8 μm pore size; Corning Costar Corporation, Cambridge, MA, United States). In brief, 1 × 10^5^ cells were plated in the upper chamber coated with Matrigel and supplemented with serum-free medium. The lower chamber was filled with a culture medium containing 10% FBS. Incubation was carried out for 48 h at 37°C, following which the non-invasive cells were scraped off with cotton swabs. The cells that had successfully translocated were fixed with 4% paraformaldehyde, stained with 0.5% crystal violet, and finally counted using an inverted microscope. MTT assay, colony formation assay, and soft agar growth assay were performed according to our previously described methods (Zhou et al., 2020). Protein structure homology modeling analysis was performed as previously described by using the online server SWISS-MODEL (Waterhouse et al., 2018).

### Evaluation of the Relationship of AS Clusters With Clinicopathological Features

Based on the identified DEAS (*n* = 371), the TCGA HCC cohort in the current study was stratified by an unsupervised consensus approach (Consensus Cluster Plus, version 3.10) (Wilkerson and Hayes, 2010). The optimal number of clusters was determined by integrating the results of the elbow method and gap statistic. The relationship between clinical outcomes and AS clusters was evaluated using log-rank test and Kaplan–Meier curves as described by Xiong et al. (2018a).

## Results

### Landscape of AS Event Profiles in HCC

**FIGURE 1:**
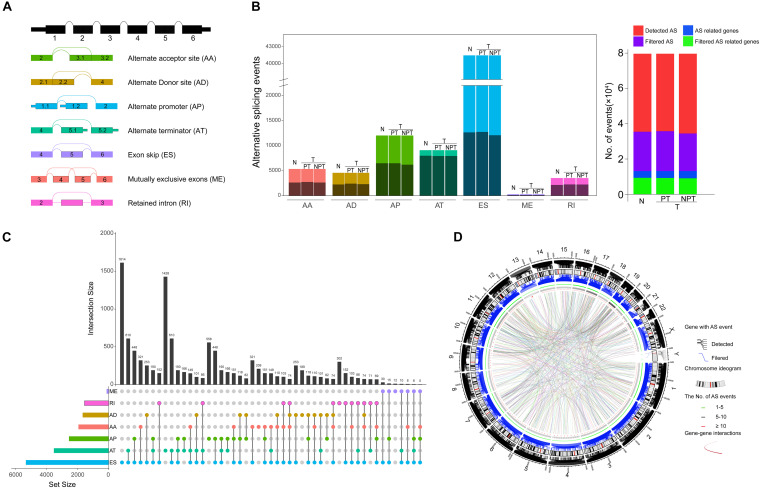
Landscape of alternative splicing (AS) events in hepatocellular carcinoma (HCC). **(A)** Diagrammatic sketch of the seven types of AS events in the present study: alternate acceptor site (AA), alternate terminator (AT), alternate promoter (AP), exon skipping (ES), mutually exclusive exons (ME), alternate donor site (AD), and retained intron (RI). **(B)** Number of AS events and the corresponding parent genes illustrated according to AS type (Left panel). The color bar represents AS events filtered by criteria. The black bar represents the corresponding genes involved in AS. Each AS type was divided into four groups based on the tissue source. N, normal tissue; T, tumor tissue; PT, paired tumor tissue; NPT, unpaired tumor tissue. Number of detected AS events, AS-related genes, filtered AS events, and the corresponding genes (right panel). **(C)** Intersection of parent genes between different AS types (*n* = 25,934) in HCC. One gene may incur up to four types of alternative splicing. **(D)** Circos plots depicting the distribution and the detailed alteration of AS events and their parent genes in chromosomes.

To systematically characterize the AS events and their clinical significance in HCC, we collected 415 RNA-Seq libraries and corresponding clinical information from 365 HCC patients (the tumor tissues and paired adjacent normal tissues from 50 patients were sequenced simultaneously). The included patients comprised 245 (67.2%) males and 120 (32.8%) females, among which 177 patients (48.5%) developed recurrence and 128 (35.4%) died of HCC. The median follow-up period was 19.5 months (range, 1–122 months). The general characteristics of these HCC patients are fully detailed in Supplementary Table S2. RNA-Seq data were associated with the clinical information of the corresponding patient *via* the TCGA barcodes. There were 50 patients with RNA-Seq data both from tumor tissue and adjacent normal tissue. According to the recommended analysis approach described in a previously published study (Ryan et al., 2016), we identified 78,878 AS events from 13,046 genes. Based on their splicing patterns, these AS events could be roughly classified into seven types: alternate promoter (AP), mutually exclusive exons (ME), retained intron (RI), exon skipping (ES), alternate acceptor site (AA), alternate terminator (AT), and alternate donor site (AD) (Figure 1A). To quantify the detected AS events, PSI values were calculated; these values measure the proportion of each detected splicing variation in all of the expressed isoforms. The expression of certain isoforms was fairly low (PSI < 0.05), and most of the AS events could not be stably detected in all of the given samples. After screening (average PSI ≥ 0.05, percentage of samples with PSI ≥ 75), a total of 25,934 AS events from 8,795 genes were obtained. We further compared the variance in quantity of AS events and the genes involved between tumor, adjacent paired normal, and unpaired tumor tissues for different splicing patterns. There were no significant differences in quantity variations; however, on average, one gene might have nearly three AS events (Figure 1B, left panel). Moreover, only 42% annotated genes in this study stably underwent AS (Figure 1B, right panel). Notably, different AS patterns may occur for a single gene. Thus, UpSet plots were used to depict the intersections between AS types. As demonstrated in Figure 1C, most of the parent genes only occurred in one type of AS event, whereas certain parent genes contained up to four types of AS event. About 81.6% of the parent genes contained two or more AS events. The arrangements of different AS types and AS events between different exons/introns may be the major reason for transcriptome diversity. In order to comprehensively depict AS event profiles in HCC, circos plots were used to visualize the relationships among AS events and the corresponding parent genes in chromosomes (Figure 1D).

### Identification of HCC-Related DEAS

**FIGURE 2:**
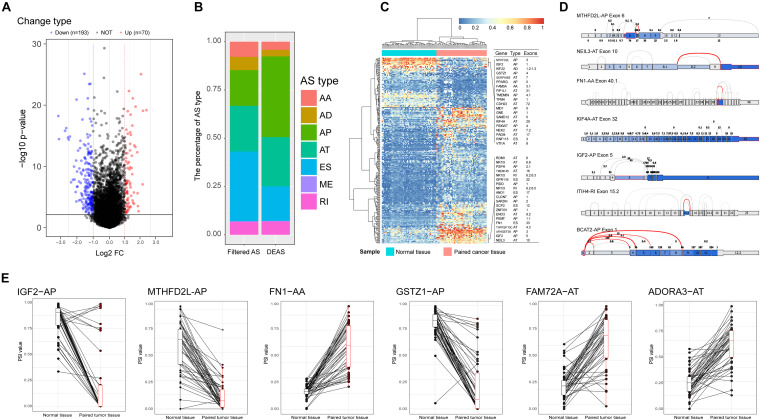
Identification of hepatocellular carcinoma (HCC)-related aberrant alternative splicing (AS). **(A)** Differences in AS events between paired HCC tissue and paracancerous tissue. Volcano plot of the differentially expressed alternative splicing (DEAS) identified in HCC; the blue and the red points represent the DEAS with statistical significance (|log(FC)| ≥ 1, adj *p* < 0.05). **(B)** Proportions of different AS types among filtered AS and DEAS. **(C)** Heat map of the DEAS. The horizontal axis shows the clustering information of samples divided into two major clusters: adjacent normal tissue (*N* = 50) and paired tumor tissue (*N* = 50); the left longitudinal axis shows the clustering information of DEAS. The gradual change of color from green to red represents the alteration of expression of DEAS from low to high. **(D)** Splice graph of some representative DEAS. The thin exon sections represent untranslated regions and the thick exon sections represent coding regions. The exons are drawn to scale, and the connecting arcs represent splice paths. **(E)** Differences in percent spliced in values of AS events between HCC and paired adjacent normal tissues.

Comparing the variations in molecular components among different pathological states using high-throughput techniques is an effective way to screen key molecules. This approach has been widely used to identify disease-related molecules in previous research (Xiong et al., 2018a, b, 2017). It is reasonable to consider that significant differences in AS events between primary HCC tissues and adjacent normal tissues may be relevant to the initiation and maintenance of HCC. In this study, the TCGA barcodes corresponding to 415 tissue samples (RNA-Seq data) were analyzed, from which AS profiles of 50 paired normal and tumor tissues were finally extracted. These paired AS profiles were used to identify DEAS. Eventually, 263 DEAS were identified from 225 genes using a threshold of |log_2_FC| > 1 and adj *p* < 0.05, including 110 APs, 47 ESs, 67 ATs, 18 RIs, nine ADs, 11 AAs, and one ME (Figure 2A and Supplementary Table S3). The top 40 DEAS are listed in Table 1. Notably, the proportion of AS types between the filtered AS and DEAS was inconsistent. The ES events accounted for 35% of filtered AS but only 17.8% of DEAS. However, the proportion of AP events rose from 18.1% of filtered AS to 41.8% of DEAS (Figure 2B). These statistical findings suggest that AS is not the result of transcription errors but a tightly regulated process. Moreover, based on the identified DEAS, the samples could be clearly separated into normal and tumor groups by unsupervised hierarchical clustering (Figure 2C), indicating that the DEAS had been reliably identified. The PSI values of DEAS events in different HCC patients are illustrated in Figure 2C as a matrix heat map. The changes in color gradient intuitively reveal the heterogeneity of HCC. A splice graph, which represents splice junctions as edges and exons as rectangular nodes, was used to visualize some of the identified DEAS (Figure 2D). Furthermore, the differences in expression of these AS events between primary HCC tissue and corresponding adjacent normal tissues are intuitively depicted in Figure 2E. Taken together, these results show that a significant variation of AS occurred during HCC initiation and maintenance, indicating that the potential role of HCC-related AS events requires further research.

**TABLE 1:** The top 40 most different alternative splicing (AS) events.

Symbol	AS type	Exons	From exon	To exon	Mean *N*	Mean *T*	Log FC	Adjusted *P-*value
**Downregulated**								
MTHFD2L	AP	3			0.538	0.112	2.266	1.393E−16
IGF2	AP	1			0.838	0.202	2.052	1.593E−16
KIF22	AD	1.2:1.3	1.1	2.2	0.328	0.080	2.030	4.948E−06
GSTZ1	AP	4			0.724	0.190	1.934	9.941E−22
SERPINB5	AT	7			0.262	0.076	1.784	2.008E−02
PPARG	AP	5			0.174	0.052	1.744	4.387E−03
FAM3A	AA	3.1	2	3.2	0.253	0.077	1.720	8.636E−05
FIP1L1	AT	31			0.295	0.091	1.702	1.393E−10
TMEM59	AP	4.1			0.497	0.164	1.598	4.574E−17
TPM4	AP	1			0.389	0.129	1.590	2.519E−11
CDH23	AT	72			0.462	0.161	1.522	1.528E−11
MID1	AP	5			0.215	0.076	1.504	2.202E−03
GNE	AP	1			0.193	0.069	1.497	6.860E−05
SAMD12	AT	5			0.181	0.066	1.451	1.522E−03
KIF4A	AT	29			0.705	0.261	1.431	2.379E−18
FBXW7	AP	4			0.295	0.110	1.416	7.699E−04
NEK2	AT	7.2			0.632	0.239	1.401	1.780E−14
PADI4	AT	17			0.436	0.165	1.399	1.843E−07
RNF115	ES	3	2	4	0.378	0.146	1.371	4.211E−10
VTI1A	AT	8			0.357	0.139	1.358	2.609E−08
**Upregulated**								
RDM1	AT	6			0.123	0.445	–1.853	1.994E−06
NR1I3	AT	6.8			0.080	0.303	–1.914	8.164E−11
PSPH	AP	2.1			0.062	0.241	–1.951	1.036E−05
TMEM145	AT	16			0.087	0.342	–1.976	7.765E−08
NR1I3	RI	6.2:6.3	6.1	6.4	0.067	0.266	–1.981	1.089E−05
GPR116	ES	22	21	23	0.118	0.473	–2.007	3.569E−18
PIDD	AP	1			0.084	0.342	–2.019	1.065E−03
NR1I3	RI	6.2:6.3:6.4:6.5	6.1	6.6	0.124	0.506	–2.030	2.135E−09
ANO1	ES	17	16	18	0.074	0.308	–2.063	5.986E−05
CLDN7	AP	1			0.060	0.263	–2.128	1.038E−06
SARDH	AP	2			0.077	0.345	–2.160	1.611E−08
SCP2	ES	12	11	13	0.051	0.241	–2.228	1.110E−06
ZNF331	AP	1			0.075	0.381	–2.342	2.586E−06
ENO3	AT	9.2			0.053	0.282	–2.415	7.736E−13
PEMT	AP	1.1			0.090	0.503	–2.484	2.117E−12
FN1	ES	33	32	34	0.051	0.294	–2.534	1.072E−12
TNFRSF10C	AT	4.3			0.075	0.466	–2.637	4.524E−14
ARHGEF39	AP	3			0.061	0.397	–2.711	1.151E−13
IGF2	AP	5			0.063	0.536	–3.081	1.829E−12
NEIL3	AT	10			0.053	0.463	–3.123	8.136E−16

### Enrichment and Interaction Analysis of DEAS

**FIGURE 3:**
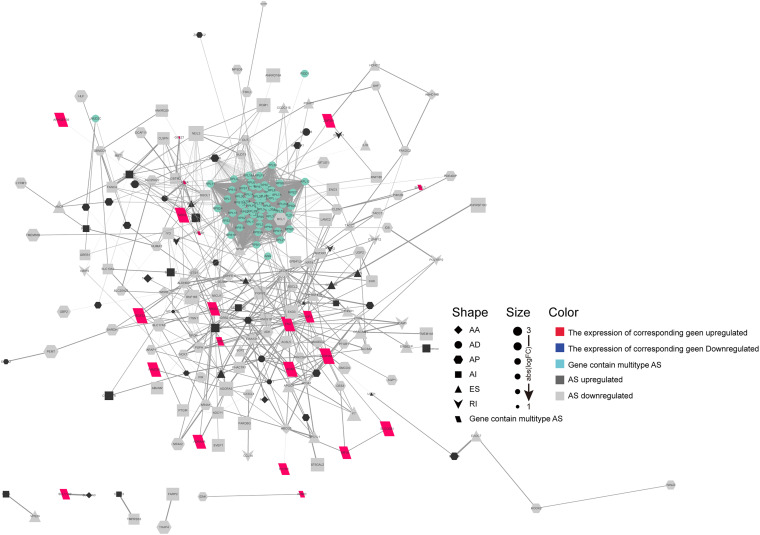
Protein–protein interaction (PPI) analysis of the identified differentially expressed alternative splicing (DEAS). Interactions of the 372 parent genes affected by DEAS. These genes were used to construct an intricate PPI network comprising 249 nodes and 514 edges. The genes are denoted as nodes in the graph, and the interactions between them are represented as edges. The shape, size, and color of the nodes, respectively, represent alternative splicing type, value of log(FC), and change pattern.

Emerging evidence indicates that AS could change a transcribed sequence directly, with effects on expression abundance or protein function. Thus, the potential biological effects of DEAS could be determined by analyzing the corresponding proteins. As shown in Supplementary Figures S2A–C, specific GO terms closely related to liver metabolism, including negative regulation of hydrolase activity, sterol homeostasis, organic acid catabolic process, and acidic amino acid transport, were significantly enriched by the parent genes of DEAS. In addition, certain KEGG pathways known to be involved in HCC were enriched, including the cGMP-PKG signaling pathway, the NF-κB signaling pathway, the mRNA surveillance pathway, and the phosphatidylinositol signaling system (Supplementary Figure S2D). These results suggest that the parent genes of DEAS are critical in the biological regulation of HCC; thus, aberrant splicing of the transcribed sequences could influence their translation and change the characteristics of the resulting proteins. Therefore, it is essential to study AS events from the perspective of PPI networks. Based on the DEAS-related genes, a PPI network was established, representing not only normal interactions but also the potential impact of AS events (Figure 3).

### Correlation Network of SFs and HCC-Related AS

**FIGURE 4:**
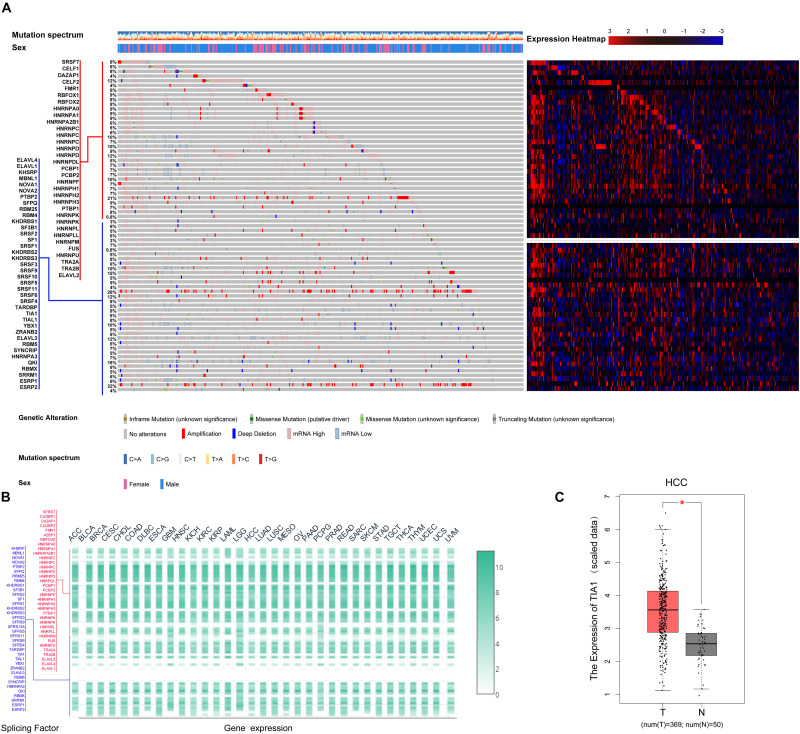
Multi-omics analysis of the 71 splicing factors (SFs) in hepatocellular carcinoma (HCC). **(A)** cBioPortal analysis of the 71 SFs in The Cancer Genome Atlas HCC patients. OncoPrint was used to produce a landscape of genomic alterations (legend) in SFs (rows) at the individual level (columns). In-frame deletions, truncated mutations, and missense mutations with known or unknown significance are shown in orange, blue, green, and gray, respectively, with one-third height. The copy number variations are annotated with the full height; amplification is shown in red and copy number loss is in blue. Heat map matrix shows the variation in SFs at expression level. The expression abundance from high to low is represented by color gradient from red to blue. **(B)** Expression of the 71 SFs in 33 tumor types. Heat map color gradient depicts the normalized expression of SFs between different tumor types. **(C)** Differential expression analysis of representative SF TIA1 in HCC. The expression of TIA1 in HCC tissue was significantly higher than that in normal liver tissue.

**FIGURE 5:**
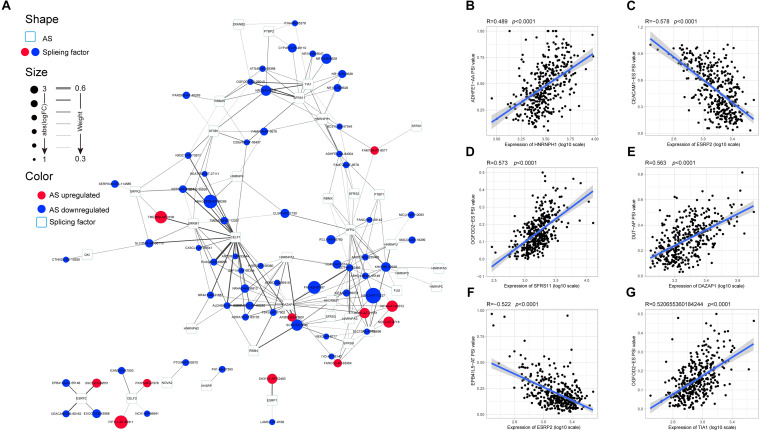
Specific regulatory network of hepatocellular carcinoma-related alternative splicing (AS) events. **(A)** Correlation network of splicing factors (SFs) and differentially expressed alternative splicing. The shape, size, and color of nodes, respectively, represent type (AS event or SF), value of log(FC), and change pattern (upregulated or downregulated). The breadth of the line represents the interaction strength. **(B–G)** Representative dot plots of the correlations between the expression of SFs and percent spliced in values of AS events.

AS events are primarily regulated by SFs, which attach to the mRNA precursor and affect the selection of exons and the choice of splicing site. Aberrant AS events in tumor tissue may be orchestrated by a limited number of SFs. For this reason, we conjecture that a few key SFs potentially regulate a large proportion of HCC-related AS events. To validate this conjecture, we first identified 71 SFs (Supplementary Table S1) by comprehensive and hand-curated screening of the literature, all of which had been previously experimentally validated (Giulietti et al., 2013). Then, the copy number variation, somatic mutations, and expression abundance of these SFs in each HCC patient were investigated using cBioPortal (Figure 4A). Visualization using OncoPrint revealed that each of the 71 SFs harbored at least three molecular alterations (Figure 4A, left panel). The most frequently affected SF was KHDRBS3, in which 96 molecular alterations were detected in 371 cases (25.8%). Partly owing to the above changes, the expression abundance of the 71 SFs showed a significant heterogeneity at an individual level (Figure 4A, right panel). The expression profiles of the 71 SFs in different cancer types also showed heterogeneous characteristics (Figure 4B). More importantly, the expression of SFs also differed between paired normal and cancer tissues of the same HCC patient (Figure 4C). Next, correlation analyses were performed between the PSI value of each DEAS event and the 71 SFs. According to the correlation coefficient (*t*-test, *p* < 0.05; |*R*| > 0.5), a splicing regulatory network was established. As shown in Figure 5A, 36 SFs were significantly correlated with 72 DEAS events, among which 60 were downregulated and 12 were upregulated. Several different AS events in the network were regulated by a single SF; in some cases, an SF had the opposite regulatory effect on different AS events (Figure 5A). We also found that the same binding site (AS event) could be competitively bound by different SFs. These observations explain, at least in part, why one gene can generate several different isoforms. Representative correlations between SFs and specific AS events were illustrated using dot plots (Figures 5B–G). For example, the expression of ESRP2 was significantly correlated with both ES of CEACAM1 (Figure 5C) and AT of EPB41L5 (Figure 5F).

### Association of DEAS With Prognosis of HCC Patients

A cross-validation method was used to evaluate the accuracy of the survival data and the clinical information. As shown in Supplementary Figure S3, stratifying patients according to the TNM stage resulted in separate Kaplan–Meier curves for both OS and DFS. These results confirmed that the survival dataset for the TCGA HCC cohort, although it contained censored data, was appropriate and informative for use in further survival analysis.

**FIGURE 6:**
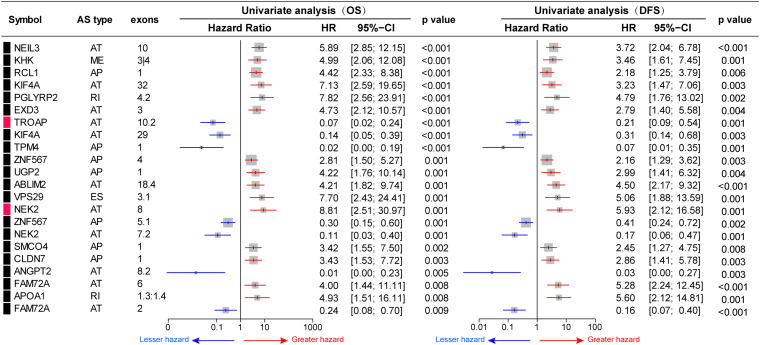
Prognostic value of differentially expressed alternative splicing (DEAS) in hepatocellular carcinoma. Part DEAS events simultaneously associated with overall survival (OS) and disease-free survival (DFS). Univariate analysis of DEAS for OS and DFS, respectively. Unadjusted hazard ratios (boxes) and 95% confidence intervals (horizontal lines) limited to alternative splicing events, with *p* < 0.01. The box size is inversely proportional to the width of the confidence interval.

**FIGURE 7:**
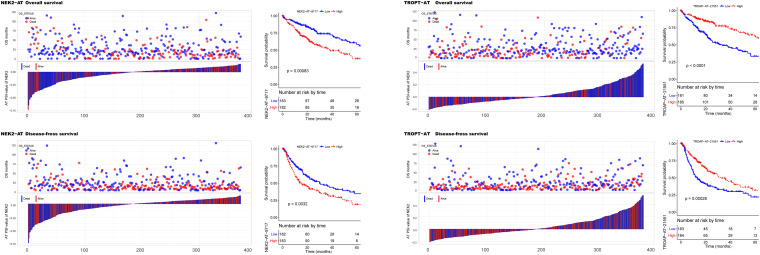
Kaplan–Meier curves for overall survival (OS) and disease-free survival (DFS) according to the percent spliced in (PSI) value of alternate terminator (AT) in NEK2 (left panel) and AT in TROPT (right panel). The PSI value distribution for each hepatocellular carcinoma patient and their corresponding survival time (OS and DFS) and survival status is shown in bar and point graphs. Kaplan–Meier curves and log-rank tests were used to compare the survival outcomes between patients with high and low PSI (median cut).

To investigate the prognostic significance of DEAS, the effect of each DEAS on survival was determined by Cox regression analysis. The HCC patients were divided into two groups according to their PSI value (median cut) of each DEAS event. According to univariate analyses, a total of 71 DEAS events (26.9%) were significantly correlated with DFS and 100 DEAS events (38.0%) were significantly correlated with OS (Supplementary Table S4). Among these prognosis-related DEAS events, 47 DEAS events were correlated with both OS and DFS (*p* < 0.05). Figure 6 shows some of the DEAS events for which the *p*-value for both OS and DFS was lower than 0.01. To demonstrate the capability of AS events for prognostic prediction, we randomly selected two prognosis-related DEAS events and used them to draw survival curves. As shown in Figure 7, according to the PSI value (median of NEK2-AT and TROPT-AT), the HCC patients could be stratified to form significant Kaplan–Meier curves by both OS and DFS survival analysis. Additionally, the DEAS events that significantly correlated with survival in the univariate analysis were further assessed by multivariate analysis. As shown in Supplementary Figure S4, there were five and six DEAS events that could be recognized as independent prognostic indicators for OS and DFS, respectively. These findings confirm that DEAS events possess not only an important biological meaning but also a potential clinical significance.

Considering the prognostic value of the above-identified AS, a prognostic model integrating multi-AS was established so that it can be easily applied to clinical practice. Based on the survival-related DEAS, a relative regression coefficient was calculated by LASSO analysis. By forcing the sum of the absolute value of the regression coefficients to be less than a fixed value, certain coefficients were shrunk exactly to zero and the most powerful prognostic marker of all the HCC survival-associated DEAS was selected, including four AS (Supplementary Figure 5A). Combining the relative expression levels of the AS in the models and the corresponding LASSO coefficients, a risk score was calculated for each patient. Obviously, patients with higher RS generally had a significantly worse overall survival than those with lower RS (*p* < 0.0001; Supplementary Figures 5B,C). As the majority of events occurred within 5 years, time-dependent ROC curves were used to assess the prognostic power based on OS at 1, 3, and 5 years, respectively (Supplementary Figure 5D).

### Clustering HCC Patients Using DEAS Associated With Prognosis

**FIGURE 8:**
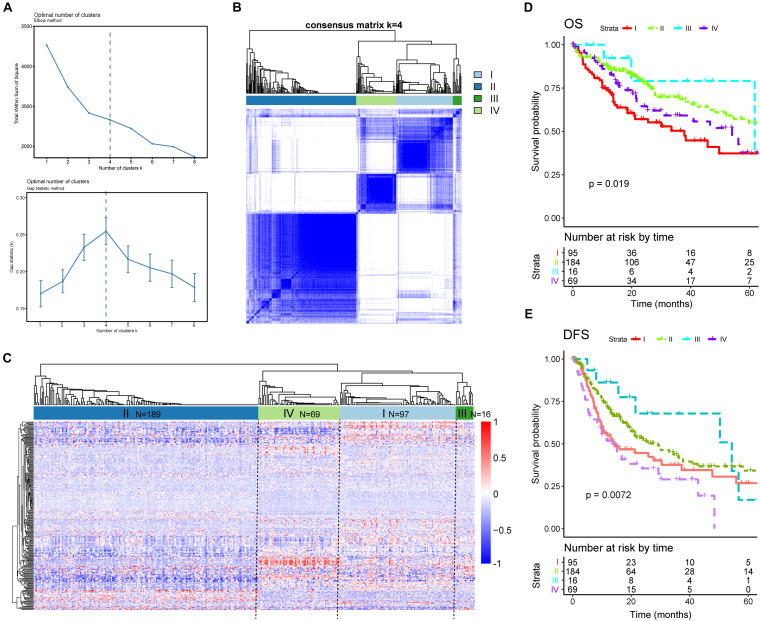
Alternative splicing clusters associated with prognosis. **(A)** Elbow and gap statistical analyses for different numbers of clusters (*k* = 2–8). **(B)** Consensus matrix heat map defining four clusters of samples for which consensus values ranged from 0 (white, samples are never clustered together) to 1 (dark blue, samples are always clustered together). **(C)** Heat map of the 263 differentially expressed alternative splicing in 371 HCC patients ordered by identified cluster showing distinct chromatism. **(D,E)** Kaplan–Meier survival analysis of patients in different clusters for both overall survival **(D)** and disease-free survival **(E)**.

Given our findings of significant heterogeneity among DEAS at an individual level, which could reflect the different outcomes of patients with HCC, we conjectured that there might exist distinct patterns of AS among different HCC patients. This hypothesis was verified using consensus unsupervised analysis based on the 263 DEAS. The optimal number of clusters was determined by combining the gap statistic and elbow method; the optimal balanced partition, as suggested, was *k* = 4 (Figure 8A). Accordingly, all the HCC patients were divided into four clusters as follows: I (*n* = 97, 26.1%), II (*n* = 189, 50.9%), III (*n* = 16, 4.3%), and IV (*n* = 69, 18.5%) (Figures 8B,C). As illustrated by the heat map, the four clusters had a significant interconnectivity, among which cluster II appeared as a well-individualized cluster, whereas there was more classification overlap among clusters I, III, and IV (Figures 8B,C). Kaplan–Meier survival analysis and log-rank test were used to evaluate the associations between prognosis and the AS clusters. As illustrated in Figures 8D,E, the stratification of HCC patients based on AS clusters showed a significant correlation with distinct patterns of survival. The variation tendency that resulted in the AS stratification between OS (Figure 8D) and DFS (Figure 8E) was basically the same. Taken together, these results indicate that splicing disorders in HCC are molecular markers of tumorigenesis and development and could be used to predict clinical outcome.

### Functional Diversity of CXCL12 Splicing Variants in HCC

**FIGURE 9:**
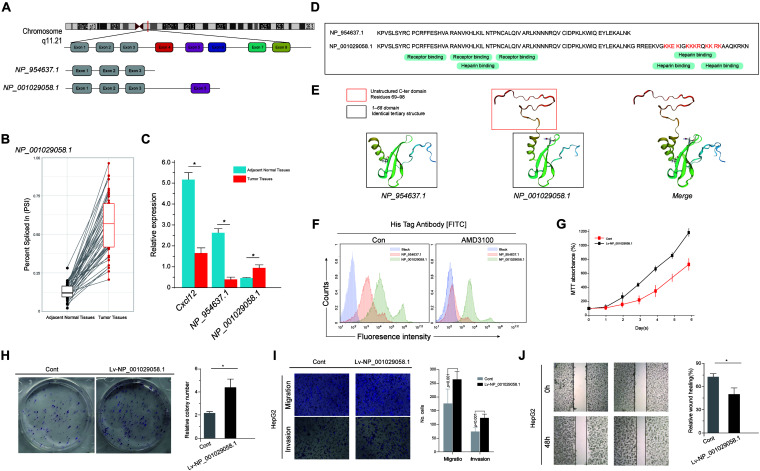
Functional diversity of CXCL12 splicing variants in hepatocellular carcinoma (HCC). **(A)** The amino acid sequences of human CXCL12 splice variants. From gene to functional protein – transcription and translation of human CXCL12 variants. **(B)** The expression level of CXCL12 at exon 5 in tumor and paired adjacent normal samples. **(C)** The expression levels of CXCL12 isoforms, NP_001029058.1, and NP_954637.1 in HCC and normal tissues. **(D)** Sequence alignment of NP_001029058.1 and NP_954637.1. **(E)** The predicted protein structure of NP_001029058.1 and NP_954637.1. **(F)** The secreted His-NP_001029058.1 protein revealed by the anti-His mAb accumulates massively at the cell surface of HepG2 cells. **(G–J)** MTT assay **(G)**, colony formation assay **(H)**, wound healing **(I)**, and Transwell assays **(J)**.

As demonstrated in the correlation network and the enrichment analysis, the differential AS events heavily influenced the chemokine activity and the NF-κB signaling pathway. HCC is a clear example of inflammation-related cancer as more than 90% of HCCs arise in the context of inflammation. Thus, we focused on these inflammation-related genes in particular (Bishayee, 2014). Among these inflammation-related genes, C-X-C motif chemokine ligand 12 (CXCL12) is an important intermediary in inflammation response and tissue homeostasis. Among the abundantly expressed transcripts of CXCL12, the alternative use of the five exons between transcripts NP_001029058.1 and NP_954637.1 was identified (Figure 9A). This alternative splicing event was labeled as CXCL12 AT Exon 5 in the current genome-wide AS analysis. CXCL12 used “Exon 5′’ to a greater extent in tumor samples, whereas CXCL12 only containing exons 1–3 was expressed much lowly in normal samples (Figures 9B,C), which means that CXCL12 AT Exon 5 could increase the expression of NP_001029058.1. According to the mRNA sequences, NP_954637.1 encodes a 68-amino-acid protein, while NP_001029058.1 encodes a 98-amino-acid protein, of which the first 68 amino acids are identical to that of NP_954637.1 (Figure 9D). Information required to run the biological function is not only stored in the amino acid sequences but also in the structure of proteins. As shown in Figure 9E, the first 68 domains of NP_001029058.1 and NP_954637.1 adopt an identical tertiary structure. Due to the additional 69–98 residues, NP_001029058.1 formed a disordered C-terminal. However, the C-terminal peptide has no major effect on the structure of the first 68 residues (Figure 9E). A previous study indicated that the intrinsically disordered domain of NP_001029058.1 could participate in the binding reaction with glycosaminoglycans. To investigate the distribution of chemokine fraction (NP_001029058.1 and NP_954637.1) associated to liver tumor cells, labeling of His-chemokine fraction expressing HepG2 using anti-His was performed. Interestingly, the secreted NP_001029058.1 protein revealed by the anti-His accumulates massively at the cell surface of HepG2 cells, which could not be reversed by AMD3100 (Figure 9F). This result suggested a strong participation of the C-terminal domain in the binding reaction with the cell surface. To further evaluate the role of CXCL12 AT Exon 5, the basic cell function of HepG2 overexpression NP_001029058.1 was explored. Both MTT assay and colony formation assay suggested that the overexpression of NP_001029058.1 promotes CRC cell proliferation (Figures 9G,H). In addition, the wound healing and Transwell assays suggested that the overexpression of NP_001029058.1 significantly increased cell migration and invasion (Figures 9I,J). These findings confirmed that CXCL12 AT Exon 5 could promote the malignant biological behavior of HCC cells.

## Discussion

AS is a basic mechanism of gene expression regulation in eukaryotes that enables the increased complexity of gene expression, facilitates a higher efficiency of transcription, and promotes protein diversity (Brett et al., 2002; Lee and Abdel-Wahab, 2016). Thus, there is reason to believe that some aberrations of AS observed in tumors may function as independent oncogenic factors, which could potentially be used as therapeutic target or prognostic biomarkers. This would explain the functional transformations of AS-related genes in cancer. In fact, the role of AS in human disease, especially malignant disease, has been widely studied in recent years (Gardina et al., 2006; Li Y. et al., 2017). However, owing to technical limitations, the function and the clinical significance of individual AS events in HCC have only been systematically studied in a few cases.

The development of RNA-Seq and its commercialized applications provides a very efficient and relatively affordable way to detect AS events at the genome-wide level (Feng et al., 2013). More importantly, RNA-Seq provides higher accuracy, increased sensitivity, and better resolution than conventional methods for AS detection (Feng et al., 2013). Detailed comparisons of quantitative PCR and RNA-Seq in differential expression analysis by the Sequencing Quality Control (SEQC) project of the MicroArray Quality Control Consortium found a good overall agreement between the two techniques (Seqc/Maqc-III Consortium, 2014). Recent evidence suggests that the performance of RNA-Seq relies on efficient algorithms and bioinformatics tools (Feng et al., 2013; Seqc/Maqc-III Consortium, 2014; Yang et al., 2015). Moreover, a study conducted by the SEQC project suggested that an appropriate algorithm and analysis strategy could improve RNA-Seq performance in many applications (Seqc/Maqc-III Consortium, 2014). In this study, SpliceSeq was used to detect AS events in the TCGA HCC cohort. Moreover, the AS events were filtered to retain the most reliable results (percentage of samples with PSI ≥ 75 and average PSI ≥ 0.05). Thus, although this study relied on RNA-Seq data alone, the results are the most reliable and robustly available for HCC.

In the present study, we integrated clinicopathological information and RNA-Seq data, obtained from a large HCC cohort, to systematically analyze AS at individual exon resolution. As far as we know, such analyses have been lacking in the past and are urgently needed, especially in the case of HCC. Through our screening and analysis, genome-wide AS events were profiled and HCC-related AS events were identified. Moreover, to investigate the underlying mechanism of aberrant AS in HCC and determine its clinical significance, enrichment analysis, network analysis, and survival analysis were conducted on the identified DEAS. Finally, a total of 25,934 AS events from 8,795 genes were screened with reliable accuracy in HCC, among which 263 AS events were differently expressed between tumor tissue and paired normal tissue. This suggested that the above-mentioned DEAS events may be associated with HCC initiation and/or maintenance. A further enrichment analysis showed that the parent genes of DEAS were likely to have vital roles in HCC. The genes that were influenced by AS events were significantly enriched in metabolism-related pathways and cancer initiation and maintenance-related pathways, including drug metabolism by cytochrome P450, nitrogen metabolism, inositol phosphate metabolism, and the cGMP-PKG signaling pathway.

In addition, the splicing correlation network provided insight into how the DEAS were regulated by key SFs. According to the results, 36 SFs were significantly correlated with 72 DEAS events, which included 12 upregulated AS events and 60 downregulated AS events. Most of the SFs were involved in the regulation of more than one DEAS, and some had opposite effects on different DEAS. In addition, the network showed that different SFs were in competition for the same binding sites (AS events), explaining at least in part why one pre-mRNA can generate several different isoforms. In other words, this observation suggested that the functional status of SFs affects the decoding of information in the pre-RNA sequence. Moreover, there were significant cell-specific and tissue-specific differences in the expression of SFs, which in turn resulted in AS specificity in different cells and tissues. For example, the expression of SFs between various solid tumors was compared, showing that the expression profiles of SFs in different tumor types were completely different. This could explain why the findings of our previously published study of DEAS in colorectal cancer were entirely inconsistent with the results of the current research. Only 27 DEAS and 39 DEAS-related parent genes were identified both in colorectal cancer and HCC. These results, combined with previous mechanism studies, further confirm that most AS events are controlled by the relative abundance and/or activity of antagonistic SFs *via* a combinatorial mechanism. The aberrant regulation of AS in tumors is frequently due to the altered expression levels, activity, or mutation of SFs instead of the mutation in the affected genes (Biamonti et al., 2014); this was clearly illustrated in the present study. According to the multi-omics analysis, the functional status of the majority of SFs among different HCC patients was altered and showed a significant heterogeneity. TIA1 is a protein-coding gene and one of the SFs in our correlation network. The product encoded by this gene is a member of an RNA-binding protein family and is involved in alternative pre-RNA splicing and regulation of mRNA translation by binding to AU-rich elements located in mRNA 3′ untranslated regions. The expression of TIA1 differed significantly between HCC tissues and paired normal tissues. Moreover, TIA1 has more than 31 RNA binding sites, suggesting that it could have an important role involving a combinatorial mechanism. In the present study, TIA1 was significantly associated with six different DEAS, among which ES of OGFOD2 was positively correlated with the expression of TIA1 (*R* = 0.521, *p* < 0.0001).

Given the importance of AS in HCC initiation and maintenance, we explored its clinical value. The diagnostic and the prognostic roles of AS in ovarian and lung cancers have been comprehensively studied (Li Y. et al., 2017; Zhu et al., 2018). Increasing evidence confirms that cancer-related AS events have potential as diagnostic and predictive biomarkers and as therapeutic targets. Wang et al. identified the loss of exon 7 of the spliced LI-cadherin gene in half of the cohort of HCC patients. After hepatectomy, the HCC patients harboring this particular AS had shorter OS (*p* < 0.01) and higher tumor recurrence rates (both *p* < 0.05) (Wang et al., 2005). Previous studies have mainly focused on individual AS events; thus, a genome-wide transcriptional analysis of survival-associated AS in HCC was urgently needed. In the present study, we systematically analyzed survival-associated AS events in patients with HCC, identifying 71 DEAS events (26.9%) that were significantly correlated with DFS (*p* < 0.05, Supplementary Table S4) and 100 DEAS events (38.0%) that were significantly correlated with OS (*p* < 0.05, Supplementary Table S4). The parent genes containing prognosis-related AS events included NEIL3, KIF4, NEK2, TROAP, and VPS29. These genes have important roles in cancer initiation and maintenance (Fu et al., 2017; Li G. et al., 2017; Hu et al., 2019) and may also be the most valuable genes for revealing the potential mechanisms of AS in HCC. More importantly, this genome-wide analysis identified many prognosis-related AS events that could represent candidate therapeutic targets for further validation.

Considering the prognostic value of DEAS, a prognostic model that integrates multi-AS was established so that it can be easily applied to clinical practice. Using this model, the prognostic risk can be assessed quantitatively at the individual level. Our data revealed that this model can successfully subdivide HCC patients into high- and low-risk groups with remarkable differences in overall survival. To the best of our knowledge, the current prognostic model is the first to evaluate the prognostic risk of HCC patients based on genome-wide AS events. More importantly, in this present study, the functional diversity of CXCL12 splicing variants in HCC was further investigated. The CXC chemokine, CXCL12, is a constitutive and broadly expressed chemokine that exerts its functions through the G-protein coupled receptor (Jung et al., 2018). From a structural viewpoint, CXCL12 has a typical chemokine fold stabilized by two disulfide bonds: it consists of a poorly structured N-terminus of 10 residues, followed by a long loop, a 3_10_ helix, a three-stranded b-sheet, and a C-terminal a-helix (Gay et al., 2007; Rueda et al., 2008). In this present study, we found that the alternative splicing event which was labeled as CXCL12 AT Exon 5 frequently occurred in HCC tissue. This AS event could change the balance of two CXCL12 isoforms (NP_001029058.1 and NP_954637.1), arising from the alternative splicing of a single gene. CXCL12 used “exon 5′’ to a greater extent in tumor samples, whereas CXCL12 only containing exons 1–3 was expressed much lowly in normal samples, which means that CXCL12 AT Exon 5 could increase the expression of NP_001029058.1. A protein structure analysis showed that the first 68 domains of NP_001029058.1 and NP_954637.1 adopt an identical tertiary structure. Due to the additional 69–98 residues, NP_001029058.1 formed a disordered C-terminal. However, the C-terminal peptide has no major effect on the structure of the first 68 residues. A previous study indicated that the intrinsically disordered domain of NP_001029058.1 could participate in the binding reaction with glycosaminoglycans (Rueda et al., 2008). Our further investigation demonstrated that CXCL12 AT Exon 5 could promote the malignant biological behavior of HCC cells. Combining the difference of protein structure and its role in promoting binding reaction, we can safely conclude that alternative splicing gives CXCL12 more functional diversity in HCC.

Aberrant AS is an intrinsic characteristic of cancer initiation and maintenance. Michael Ladomery considers it to be another hallmark of cancer (Ladomery, 2013). If aberrant AS did reflect the underlying molecular mechanism of HCC, the HCC patients could be stratified with distinct clinical outcomes by variations in DEAS expression at the individual level. To verify this hypothesis, we performed consensus on an unsupervised analysis based on the 263 DEAS. According to our results, the stratification of HCC patients based on HCC-related DEAS was closely correlated with distinct patterns of Kaplan–Meier curves. The variation tendency that resulted from AS stratification between OS and DFS was basically the same. These findings show that there is considerable heterogeneity in AS events across HCC, which could influence clinical outcomes.

Although most AS events were excluded by strict quality controls, these allowed us to be confident that AS events are ubiquitous, rather than transcription, errors in HCC patients. After a series of bioinformatics analyses, the PSI values of 263 AS events were screened, showing significant differences between normal and paired HCC tissue samples. Furthermore, an enrichment analysis indicated that the parent genes of these AS events may have important roles in liver metabolism and cancer-related pathways. The splicing network also shed light on the underlying mechanism of regulation of HCC-related AS events by SFs. The prognosis-related AS events, which are very important for understanding the mechanisms of AS in the initiation and the maintenance of HCC, suggest new therapeutic targets for further study.

## Data Availability Statement

The datasets generated for this study can be found in the data that support the findings of this study are openly available in TCGA SpliceSeq (https://bioinformatics.mdanderson.org/TCGASpliceSeq/PSIdownload.jsp). The datasets used and/or analyzed during the current study are available from the corresponding author on reasonable request.

## Author Contributions

YX and JL contributed to the conception and the design of this study. YX developed the methodology. YX, GY, KW, and HZ contributed to the acquisition of data (provided animals, acquired and managed patients, provided facilities, etc.). YX, JX, ZL, QL, JS, and WL contributed to the analysis and interpretation of data (e.g., statistical analysis, biostatistics, and computational analysis). YX, ZL, TT, WL, and MR contributed to the writing, review, and revision of the manuscript. JL and YX provided administrative, technical, and material support (i.e., reporting and organizing data and constructing databases). All authors contributed to the article and approved the submitted version.

## Conflict of Interest

The authors declare that the research was conducted in the absence of any commercial or financial relationships that could be construed as a potential conflict of interest.

## Footnotes

1 https://portal.gdc.cancer.gov/

2 http://gdac.broadinstitute.org/

## Supplementary Material

FIGURE S1 Flowchart for profiling the alternative splicing of hepatocellular carcinoma in a large-scale RNA-Seq dataset.

FIGURE S2 Enrichment analysis of the parent genes of differentially expressed alternative splicing (DEAS). **(A–D)** Gene Ontology (GO) and Kyoto Encyclopedia of Genes and Genomes (KEGG) pathway analyses of the parent genes of identified DEAS. The vertical axis represents GO or KEGG pathway annotations. The horizontal axis represents the number of genes assigned to the corresponding annotation. **(A)** Biological process, **(B)** cellular components, **(C)** molecular function, and **(D)** KEGG pathways.

FIGURE S3 Kaplan–Meier estimates of overall survival (OS) and disease-free survival (DFS) stratified by TNM stage in The Cancer Genome Atlas hepatocellular carcinoma cohort. **(A)** OS. **(B)** DFS. The differences between the two curves were determined by a two-sided log-rank test. Overall log-rank test, *p* < 0.0001.

FIGURE S4 The differentially expressed alternative splicing events that present with independent prognostic value on survival. Hazard ratios (boxes) and 95% confidence intervals (horizontal lines) limited to alternative splicing events with *p*-value < 0.05. The box size is inversely proportional to the width of the confidence interval.

FIGURE S5 Construction of the integrated prognostic model. **(A)** LASSO coefficient profiles of the 12 survival-related alternative splicing. **(B)** Prognostic model analysis of the patients. The distribution of risk score: patients’ survival time and status. The black dotted line represents the cutoff dividing the patients into low-risk and high-risk groups. Heat map of the RNAs in the prognostic model. **(C)** Kaplan–Meier estimates of overall survival for prognostic model. **(D)** Time- dependent receiver operating characteristic curves.
