# Supplementary figures and images for: Genome-Wide Transcriptional Analysis Reveals Alternative Splicing Event Profiles in Hepatocellular Carcinoma and Their Prognostic Significance
Source: Front Genet. 2020 Aug 11;11:879. doi: 10.3389/fgene.2020.00879 (PMC7432180; doi:10.3389/fgene.2020.00879)

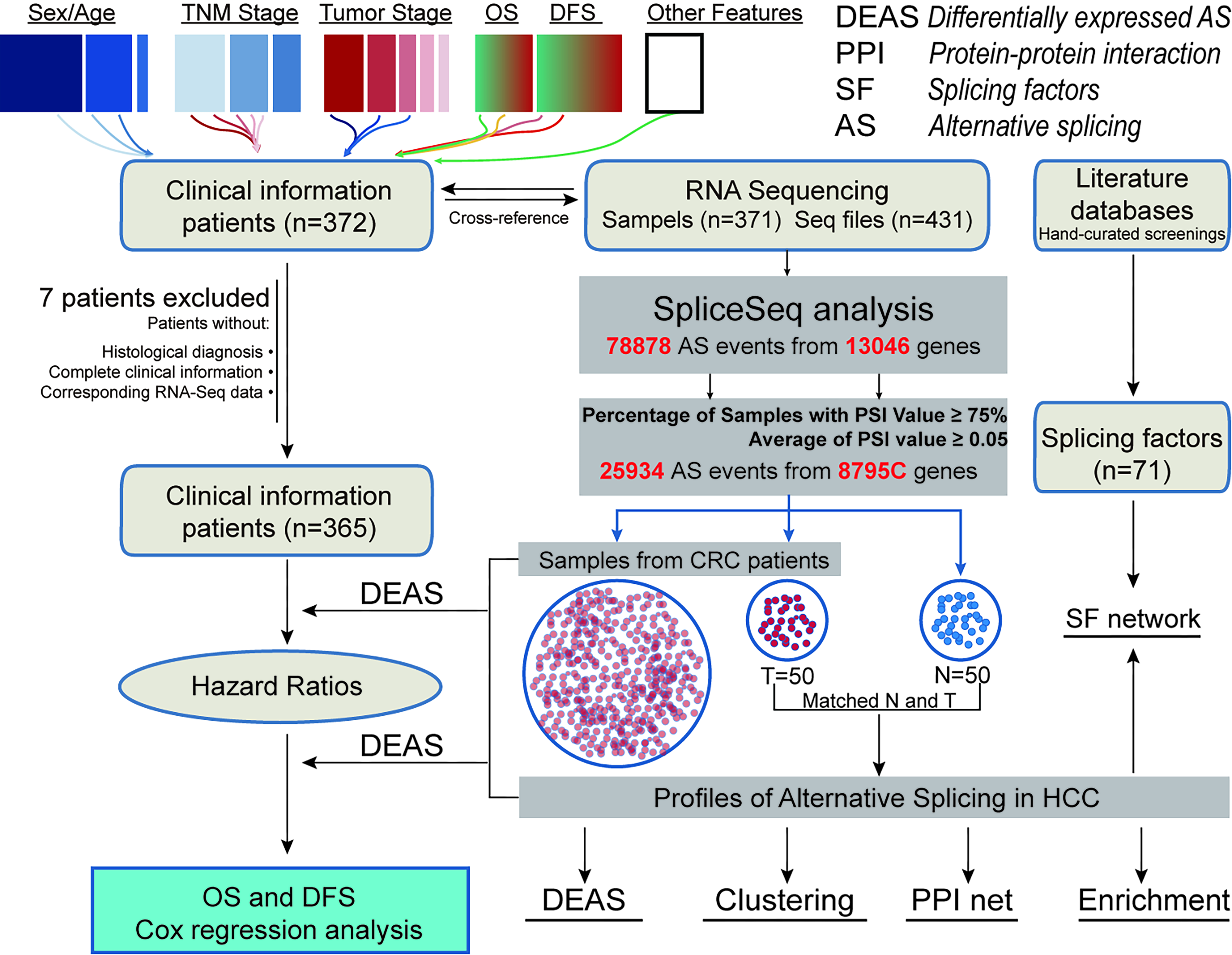

Supplement: FIGURE S1 — Flowchart for profiling the alternative splicing of hepatocellular carcinoma in a large-scale RNA-Seq dataset. [file Image_1.TIF]

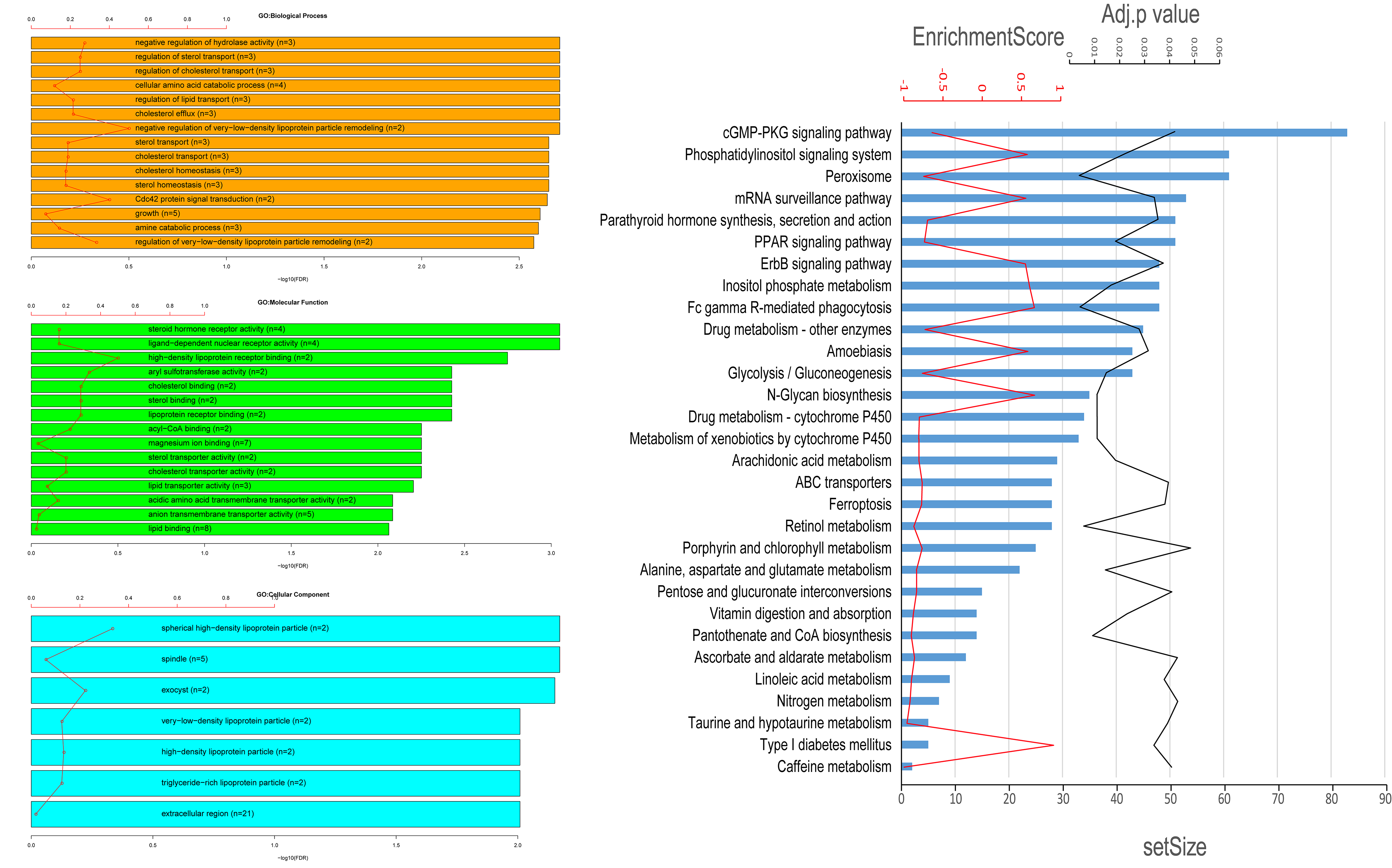

Supplement: FIGURE S2 — Enrichment analysis of the parent genes of differentially expressed alternative splicing (DEAS). (A–D) Gene Ontology (GO) and Kyoto Encyclopedia of Genes and Genomes (KEGG) pathway analyses of the parent genes of identified DEAS. The vertical axis represents GO or KEGG pathway annotations. The horizontal axis represents the number of genes assigned to the corresponding annotation. (A) Biological process, (B) cellular components, (C) molecular function, and (D) KEGG pathways. [file Image_2.TIF]

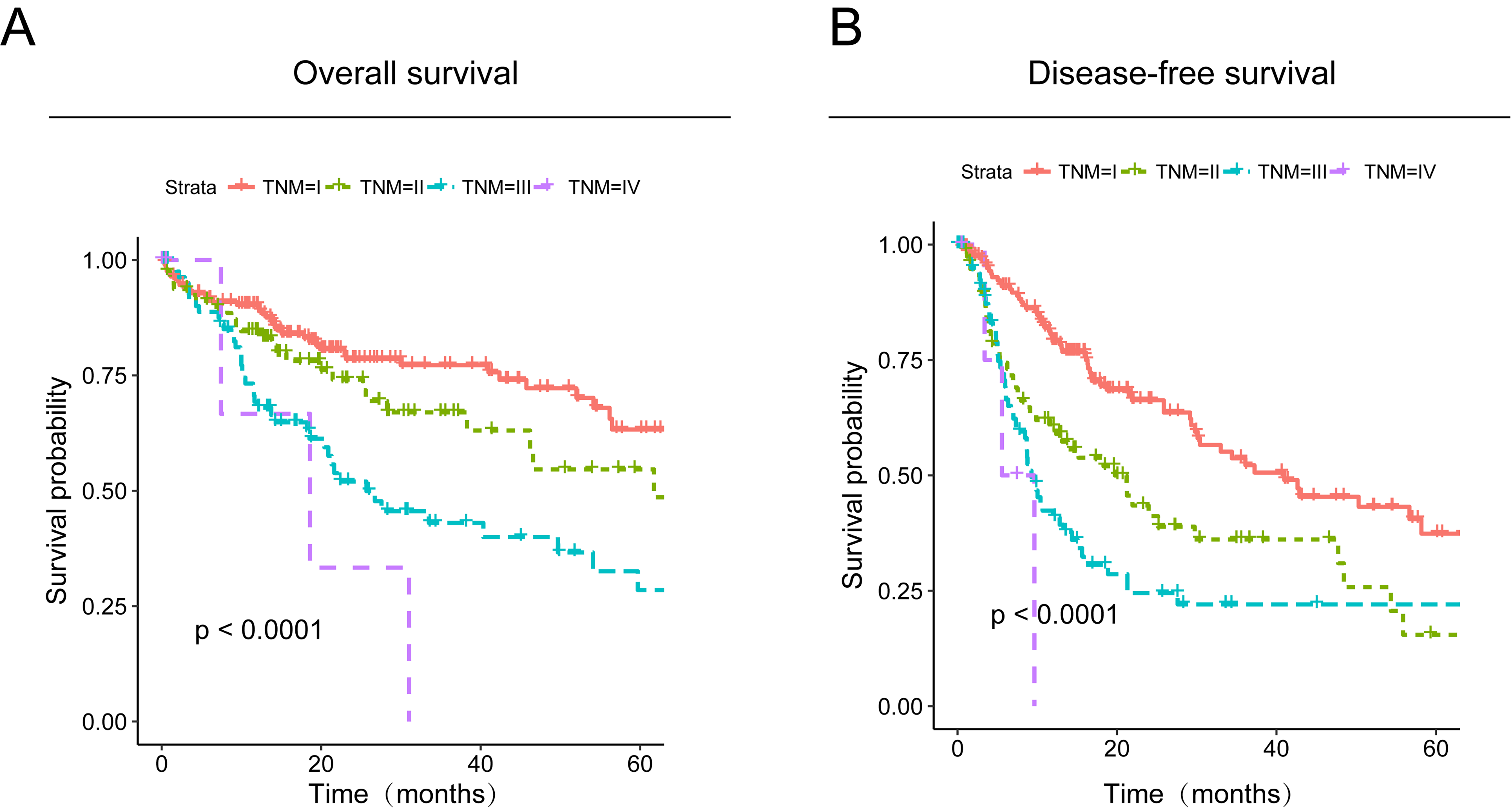

Supplement: FIGURE S3 — Kaplan–Meier estimates of overall survival (OS) and disease-free survival (DFS) stratified by TNM stage in The Cancer Genome Atlas hepatocellular carcinoma cohort. (A) OS. (B) DFS. The differences between the two curves were determined by a two-sided log-rank test. Overall log-rank test, p < 0.0001. [file Image_3.TIF]

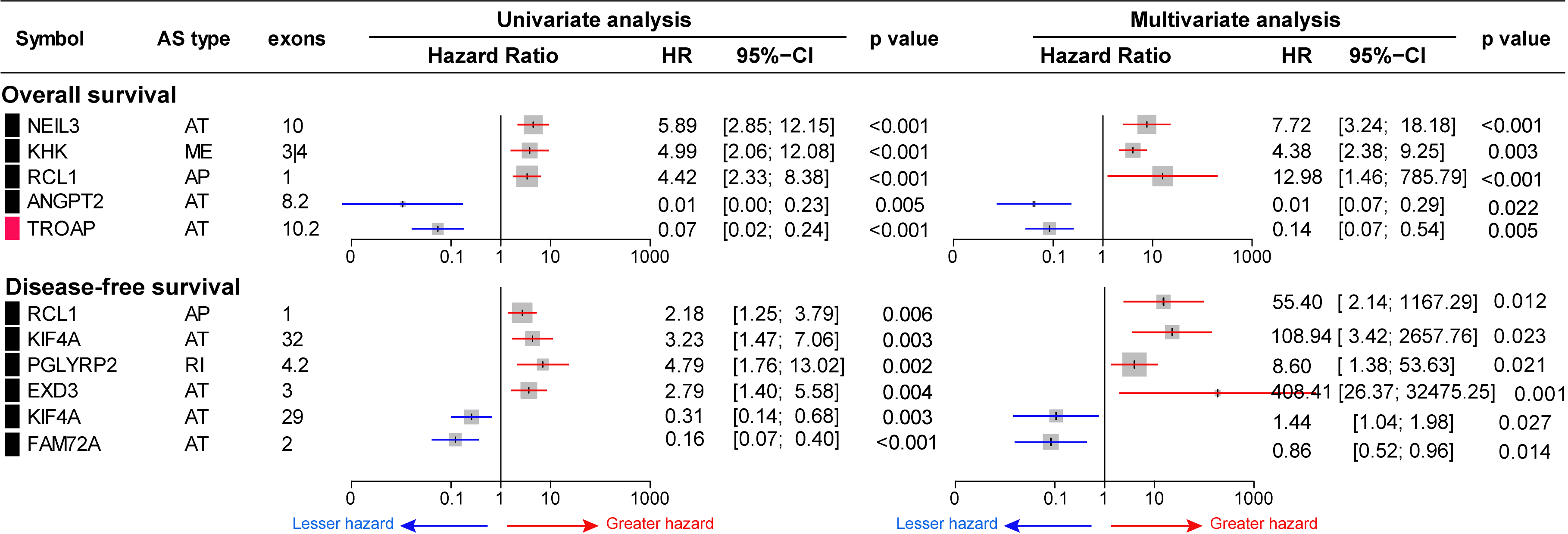

Supplement: FIGURE S4 — The differentially expressed alternative splicing events that present with independent prognostic value on survival. Hazard ratios (boxes) and 95% confidence intervals (horizontal lines) limited to alternative splicing events with p-value < 0.05. The box size is inversely proportional to the width of the confidence interval. [file Image_4.TIF]

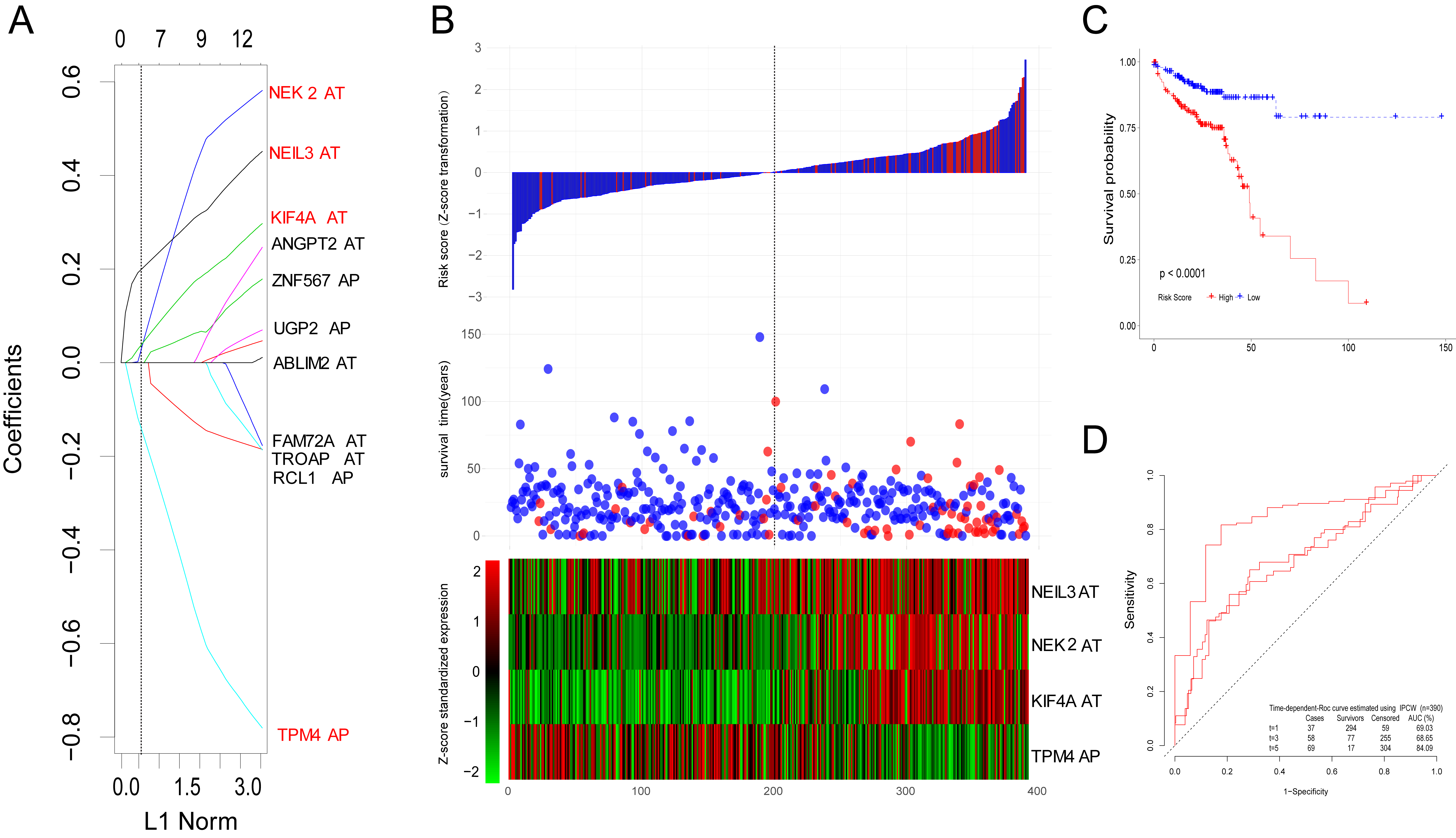

Supplement: FIGURE S5 — Construction of the integrated prognostic model. (A) LASSO coefficient profiles of the 12 survival-related alternative splicing. (B) Prognostic model analysis of the patients. The distribution of risk score: patients’ survival time and status. The black dotted line represents the cutoff dividing the patients into low-risk and high-risk groups. Heat map of the RNAs in the prognostic model. (C) Kaplan–Meier estimates of overall survival for prognostic model. (D) Time- dependent receiver operating characteristic curves. [file Image_5.TIF]
